# Advancing the state-level tracking of evidence-based practices: a case study

**DOI:** 10.1186/s13033-019-0280-0

**Published:** 2019-04-10

**Authors:** Sarah Cusworth Walker, Georganna Sedlar, Lucy Berliner, Felix I. Rodriguez, Paul A. Davis, Savannah Johnson, Jessica Leith

**Affiliations:** 1 Department of Psychiatry and Behavioral Sciences, University of Washington, 1100 NE Campus Parkway, Box 358015, Seattle, WA 98105 USA; 2 Harborview Center for Sexual Assault and Trauma, 401 Broadway, Seattle, WA 98104 USA; 3 Washington State Health Care Authority, 626 8th Ave SE, Olympia, WA 98501 USA; 4 Duke University, 2127 Campus Drive, Box 90065, Durham, NC 27708 USA

**Keywords:** Implementation, Evidence-based practice, Evidence-based policymaking, Common elements, Learning system, Children’s mental health, State policy

## Abstract

**Background:**

Despite a sustained focus by policymakers and researchers on improving the standard of clinical care in public mental health services, the use of evidence-based practice remains low. Among other challenges, this reflects the difficulty of translating clinical research into useable policy that can be feasibly funded and monitored by state or large healthcare systems.

**Case presentation:**

In this paper we present a case study of Washington State’s strategy for monitoring the use of clinical elements at the session level for all Medicaid-funded children’s mental health services. The implementation of this strategy reflects policy actions to promote effective practice while also actively influencing multiple other levels of the implementation ecology. The approach is informed by the Policy Ecology Framework, the Consolidated Framework for Implementation Research, the evidence-based policymaking literature, and common ontology and clinical elements models.

**Conclusions:**

We found the strategy developed in Washington State to be a feasible method of collecting session level information about the use of effective clinical mental health practices. In addition, the approach appears to be having influence on multiple layers of the implementation ecology that could be explored through further study.

## Background

Nearly 40% of children will experience a psychiatric disorder before age 18 [1] and medical costs for children’s mental health are one of the top five most expensive healthcare services in the US [2]. The extent of population mental health need and expense is a significant public health issue; however data on the use of effective mental health treatment and outcomes is nearly nonexistent. The limited available data suggests that tested psychosocial treatments, e.g., evidence-based practices (EBP), are sparse and may be decreasing [3] despite considerable efforts in multiple states to invest and promote these services [4]. Among multiple barriers to utilization is the difficulty of paying for the training, consultation and fidelity monitoring packages involved in implementation. While some states have had significant success in funding specialized services, no public mental health system has succeeded in establishing EBP as an expected approach of routine clinical care statewide. Achieving this will require innovative approaches in treatment development, organizational strategies and policy climates supportive of learning approaches to performance monitoring and funding [5, 6].

The barriers involved in instituting evidence-based practices across large service systems are complex and well-documented. These include low political will, concerns about cultural responsiveness, funding challenges, lack of emphasis in graduate training, high workforce turnover in public mental health, and limited organizational capacity to adopt innovations [7–11]. In their paper describing New York State’s approach to scaling up EBPs, Hoagwood et al. [12] describes the costs of implementing EBPs to fidelity as “enormous” and recommends that state level efforts also support policy and organizational strategies as a complement to direct training and consultation initiatives [12]. Current implementation science frameworks tend to describe the policy context, or ecology, as a dimension that can support or impose research-based products, [5, 13] recognizing the powerful role policy plays in creating a funding and regulatory environment that can spur the adoption of EBPs. This may include setting mandates, providing incentives, and directly funding education, training and consultation [14]. In this paper we use a case study approach to describe how a legislative agenda created a series of cascading effects on multiple levels of the implementation ecology. We argue that a key catalyst for turning legislative intent into meaningful impact was the creation of a system to track EBP delivery statewide. We discuss the potential of this approach for performance monitoring and allocating enhanced payments for value-based care.

### Implementation frameworks

In their 2008 paper, *Towards a policy ecology of implementation of evidence*-*based practices in public mental health settings*, Ragahavan, Bright and Shadoin note that focusing on mandating EBP use or narrowly focusing on individual change within organizations is unlikely to result to broad and sustainable EBP uptake (pg. 26) [5]. To encourage a focus on the systems governing innovation, their proposed framework suggests four levels of intervention at the policy level: Provider organization, Regulatory or purchaser agency, Political and Social. The authors outline strategies that can be taken at each level to promote EBP which include, among others, flexible and enhanced reimbursement, prior authorization, mental health parity and anti-stigma campaigns. A particularly useful contribution of this framework is the recognition that policy level implementation strategies are likely to be highly idiosyncratic. As implementation moves towards the outer level, the number of factors that must be incorporated into a successful strategy quickly expands. This complexity is highlighted, for example, in Powell et al’s 2016 paper describing the City of Philadelphia’s multi-pronged effort to influence the outer context of implementation [13]. This effort involved a number of strategies at every level of policymaking each of which was also tailored to the practice, funding and political realities of the local context. Given this complexity, it is unsurprising that efforts to study and influence implementation from the policy level are less well represented in the implementation literature.

More comprehensive implementation frameworks are helpful in guiding and understanding the impact of these policy level strategies. The Consolidated Framework for Implementation Research (CFIR), for example, is a well-known model that provides a useful taxonomy outlining the levels of implementation, including policy, that influence uptake and sustainability of new practices. The framework was proposed in 2009 as an updated synthesis of healthcare implementation and innovation diffusion models and comprises five major domains: the intervention, the inner and outer setting, the individuals involved and the process of implementation. These levels echo previous work characterizing the importance of the context of implementation, characteristics of the intervention and the process of implementation for managing organizational change [15–17]. For our purposes, the CFIR was a helpful framework for organizing the hypothesized effects of the Washington State strategy on multiple layers of the implementation ecology as will be described in more detail below.

### Evidence-based policymaking

Another area that provides context for the effort in Washington State is the literature on use of research in policy. As described in an influential text on evidence-based policymaking, researchers who attempt to bridge the research to policy gap as intermediaries “see policymakers as their primary clients. In addition to producing knowledge, they also see their role as translating extant research and analysis in ways that enhance their utility for those doing public policy” [18]. Similarly, a report by the National Research Council, Committee on the Use of Social Science Knowledge in Public Policy, [19] noted the evolution of evidence-based policymaking over the last 40 years, which has shifted from providing education, technical assistance and list-making to active engagement in shaping the decision-making climate.

This is important because it highlights the need for adaptability and a greater emphasis on what Damschroder et al. [20] described as the core elements vs. adaptable periphery of studied interventions at all levels (intervention, implementation and policy). As the scholarship on research and policy notes, research is only one source of information amongst many other competing demands, including constituent or leadership priorities, capacity to reform, the cost–benefit of change and political will [19, 21, 22]. To maintain focus and political will for the use of research in policymaking, brokers of this process need to actively synthesize and interpret research findings so core features are preserved in adaptations. Developing policy strategy to support the end goal of a client receiving a service that can be reasonably defined as “evidence-based” will necessarily involve conversations about how evidence-based is defined [23, 24] as well as what monitoring infrastructure is needed to feasibly track use [25].

The field of implementation science recognizes the importance of the policy environment in supporting or deterring system change efforts to promote the use of evidence-based practices. While policy is highlighted in multi-layered implementation frameworks, such as the CFIR, little is known about how policy can directly influence other elements of the implementation ecology. We believe our efforts in Washington State can provide an illustrative example of how policy, organizational and intervention level effects influence each other reciprocally in this ecosystem.

## Case presentation

The Reporting Guides for Evidence-Based Practice for Children’s Mental Health emerged in response to the passage of HB2536 in Washington State in 2012 and now in statute as Chapter 43.20C RCW. The bill directed the three child-serving departments (mental health, juvenile justice, child welfare) to “substantially” increase investments in evidence-based practice and required each state department to submit an annual report on its investment for the subsequent 5 years. The first EBP list developed for reporting was taken from the Washington State Inventory of Evidence-based, Research-based and Promising Practices (WSIPP) [24, 26] also developed as part of the 2012 legislation. Reporting codes were assigned to each of the programs listed on the inventory and agencies were directed to use the assigned codes to report the use of EBPs per session. At this stage of implementation, the state provided no other reporting guidance for required training, consultation or documentation.

Ambiguity about the reporting requirements discouraged providers from using the codes, resulting in very low reported rates and distrust in the accuracy of data (Endler, personal communication). To address this concern, the state requested that the University of Washington-based Evidence Based Practice Institute develop quality guidelines for reporting and tracking use. The state and EBPI were faced with how to track EBPs under three conditions: (1) infrequent real world use of EBP manuals to fidelity; (2) no resources for the expert review of therapist competence on a large scale; and (3) needing to track use through a billing code tied to a unique session. Further, to address previous concerns about ambiguity, the guidelines needed to be very concrete about what constituted sufficient EBP practice. The developed method for tracking use addressed these constraints by, respectively, (1) using a treatment family and common elements ontology to justify the use of discrete clinical elements in session as reportable EBP; (2) accepting approved training and case note documentation as good enough proxies for competence; and (3) providing specific language for documentation to overcome provider concerns about the accuracy of self-report. We elaborate on the literature supporting these approaches below.

### Training and documenting practice as proxies for assessing use

Without an external method of determining the clinician’s actual use of skills, we determined that exposure to an interactive active training would provide the first safeguard for ensuring competency. Requiring training was fairly straightforward as it is the most common method for teaching new concepts and clinical skills [27]. While reading a manual or participating in a self-guided online training substantially lowers the barrier to entry for learning new materials, the effectiveness of these methods in supporting the competent use of new skills has little or mixed support in the available literature. For example, a systematic review of the effectiveness of the dissemination of printed clinical guidelines on practice did not substantively improve patient outcomes [28]. Having the opportunity to practice skills and receive feedback appears to increase the likelihood of skills transfer. This is supported by the general literature in medical training, with high quality simulation approaches showing the best effects in supporting clinician skills and patient outcomes [29].

However, a complication of requiring training, and also opening the allowable training to non-proprietary, common elements-based models, is that the field lacks empirically-informed standards for length, format and post-training support. The length and format of trainings vary widely among purveyor-led EBPs. Training time among programs range from 40 h to a few days, [27], or may be purveyed entirely through self-guided media platforms [30, 31]. The same complication applies to the length of consultation available to therapists following training, with some models requiring indefinite consultation and some not requiring or offering any. While waiting for more empirical guidance in this area [32], we opted for not specifying any specific length of training time. Instead, the guides indicate the need for interactivity and some objective assessment of skill. Consultation requirements are left to the discretion of the training entity or proprietary program.

### Clinical treatment families and common elements

The concept of clinical treatment families is central to the organization and documentation requirements in the Reporting Guides. The concept of treatment families is promoted by influential scientific groups attempting to develop frameworks to support more rapid synthesis and translation of psychosocial intervention science [33–35]. A treatment family encompasses programs that share a theory of change and practice elements. It is a method of synthesizing intervention science to unpack the black box of single intervention studies. Using treatment families can help create a shared language amongst agencies and providers and clarify how specific practice elements are being used to address specific clinical needs [34].

Chorpita et al. were the first to attempt a large scale disaggregation of the clinical components of child and youth psychotherapy across multiple evidence-based programs [33]. Updated in 2009, the *Distillation and Matching Model*, [33] was designed to provide a detailed description of common clinical strategies as they occurred in families of treatments identified by the intended area of clinical need (e.g., anxiety, externalizing behavior). In their formulation, a practice element is a discrete clinical technique (e.g., relaxation, time out) as part of a larger intervention. Practice elements were coded directly from over 200 controlled trials in which the tested program was superior to the control. Definitions of elements were informed by practice experts and showed an acceptable inter-rater reliability (k = .76) [33].

A related effort is being led by the Society for Behavioral Medicine Interest Group (SIG) and the Theories and Techniques of Behavior Change SIG [36]. This effort is developing an *ontology¸* or common language, for behavioral change across health sciences. Modeled off the Human Genome project (i.e., the Gene Ontology), the effort aims to develop a taxonomy of controlled vocabulary related to behavior change principles and outcomes to facilitate the sharing of knowledge across studies. This is intended to result in hierarchical structures of classes in which, for example, the class of “Goals and planning” might include multiple behavioral indicators such as “Goal setting behavior,” which itself is made up of lower level components such as “setting a goal in an appropriate time frame.” Coding intervention studies and results using a common ontology could result in more rapidly synthesized results for developing new interventions and policy approaches to support implementation [34].

In addition to providing conceptual clarity, a common elements framework may also support better clinical practice. As *ad hoc* adaptation of programs is the norm following training and consultation, [37] an orientation to the theory of change and empirical support for discrete elements in protocols may prevent fidelity drift. For example, a study of therapist practices after receiving significant training and supervisory support for a clinical trial found that therapists were infrequently using the most effective components of CBT for anxiety (exposure) and depression [38]. This common elements approach is also advocated in the Institute of Medicine, Committee on Developing Evidence-Based Standards for Psychosocial Interventions for Mental Disorders [34]. In this monograph, the committee recommends using a common language approach to clearly communicate about the components of programs in order to reduce confusion about what practices are effective and in what contexts they are effective [34].

The use of select EBP practice elements appears to overcome frequently cited concerns about EBPs being too rigid and failing to meet the complex needs of families in the real world [39–41]. Providers may be more amenable to adoption of EBPs if these treatments are broken down into specific practice elements in order to provide more flexibility, gain the ability to retain clinical judgment and maintain an individualized approach when treating patients.

### Guidance for documentation and self-report

To use an EBP code, a therapist documents their practice in two places within routine notes. Documentation occurs first in Treatment Plans and then in the Progress Note for individual sessions. The two levels of documentation are expected to establish that the provider is aware of the essential or most active clinical elements of a treatment family while also being allowed to use a broader list of common elements in any individual treatment session. The Guides require that the provider indicate the treatment family or proprietary EBP program and the intention to use at least one essential element in the proposed course of treatment. After indicating intent to use an essential element in the treatment plan, the provider is free to use any allowable elements in the individual treatment sessions.

Essential and allowable elements differ from each other by (1) how often they are included in empirically supported treatments and (2) as how unique they are across treatment families. The development team identified these components by first examining the 2005 and 2009 core component reviews conducted by Chorpita et al. and Chorpita and Daleidan to identify all components present in 50% of programs found to be effective under each treatment family [33, 42]. This comprised the first working list of allowable elements that could be used in progress note documentation. We then took components present in 80–100% of effective programs as our first pool of essential elements. From this pool, we removed elements that were present in all or most treatment families (e.g., psychoeducation), retaining elements that defined the treatment family in unique clinical approach (e.g., exposure). The resulting lists were reviewed internally and externally by two PhD level psychologists and two Master’s level therapists trained in evidence-based models of child and adolescent psychotherapy. To support user-friendly language, we cross-walked the components pulled from the Chorpita and Daleiden papers with the common elements used in a widely disseminated common element-based training in Washington State [42, 43]. The final guidelines provide specific wording for documenting use in treatment plans and progress notes. This wording reflects the crosswalk from an EBP, the associated treatment family, and the clinical elements used in the specific session.

The practical directives provided in the Reporting Guides reflect the policy mandate to count the state’s investment in EBPs while also reciprocally influencing the implementation ecology. In adopting a definition of EBP that reflected the need to count practices, the Guides have subsequently shifted site level practices that are influencing the inner and intervention levels as described below.

### Intersection with multiple layers of the implementation ecology

Washington State developed a statewide system to track EBP service delivery in support of a legislative mandate to increase use. To overcome the challenges inherent in tracking EBP at this scale, guidance for reporting made use of a treatment family, common elements framework and designated approved training entities. These decisions had reciprocal effects on the implementation climate beyond the simple intent of increasing use. Using the CFIR and Policy Ecology frameworks, we outline the observed and potential impacts of this strategy in Washington State.

### Outer layer

All of the strategies in the policy ecology framework are in the outer layer of the CFIR. These include flexible reimbursement, regulations, contracting, data collection, and legislation (Table 1). While the policy ecology framework does not suggest how the various activities at the policy level may be related to lower levels of the implementation ecology, the experience in Washington State suggests a series of cascading effects. Legislation requiring annual reporting of EBP use created an environment that supported EBP without specifying penalties. This signaled the state’s interest in EBP while avoiding some of the risks noted in the literature around imposed use [5]. As a result, the executive branch had to develop a system to track EBPs but were not given enough funding to track use using expert review and validation. In addition, the legislative mandate to use the state inventory deferred questions about how to define EBP to in-state intermediaries (UW/EBPI and Washington State Institute of Public Policy). This allowed the intermediaries to construct solutions to address the unique circumstance of Washington State. This, in turn, allowed the intermediaries to consult with expert clinician research and trainers when devising a state tracking system that would be responsive both to policy, research and practice. Consequently, we observed a reciprocal effect between a policy and practice such that the need to devise a method of tracking EBP (data collection) influenced the subsequent definition of EBP (legislative intent).

**Table 1:** Crosswalk of implementation frameworks and the Washington State strategy

Consolidated framework	Policy ecology framework	Washington State
Outer setting	Provider organization	Hypothesized effects
Patient needs and resources	Flexible and enhanced reimbursement	Modifier codes tracking provides relatively low burden monitoring for enhanced reimbursement
Cosmopolitanism		
Peer pressure		
External policy and incentives		
	Regulatory or purchaser	Contracts requiring the reporting of EBP increases awareness and motivation
	Changing contracting	Benchmark reporting creates transparency and social pressure to implement
	Collect data	Modifier codes create system wide data tracking method on practices
	Enabling legislation	Legislation requiring a report of investment and increased use supported a climate of EBP use
Inner setting		
Structural characteristics		Required reporting shifts supervision focus to core elements
Networks and communications		Workshop and technical assistance on using the guides expands the network of communication
Culture		Documentation of use in treatment plan and notes increases attention to adherence within the organization
Intervention characteristics		
Intervention source		Eligible training programs are assessed based on their incorporation of evidence-based core elements
Adaptability		Core elements conducive to the adaptable periphery
Trialability		The guides attempt to reduce complexity by crosswalking name brand language with core concepts
Complexity		Focus on core elements simplifies therapeutic concepts
Cost		The guides support reduced costs by allowing different purveyors to enter the market

### Inner layer

The CFIR inner layer describes organizational characteristics that affect implementation and organizational learning. Of the five elements in this layer, the statewide system of EBP tracking is expected to have an effect on at least two other elements: (1) Increased networks and communications, and (2) culture. Networks and communications relates to an organization’s connectedness with external sources of information and possibly innovation. In receiving the Guides, every agency and individual provider with a Medicaid contract is being exposed to the most effective programs and effective clinical elements across programs. For providers not already connected to universities or following clearinghouses, the Guides may be their sole source of exposure to evidence-based practices. The Guides are also expected to shift internal culture by providing specific language to use when documenting practices in treatment plans and notes. While efforts are underway to systematically study these changes, there is anecdotal evidence that the guides are beginning to shift supervision practices. Supervisors are reporting that they are using the Guides to shape clinical supervision practices, and in at least one medium sized community mental health agency, clinician treatment plans and notes are approved only if they conform to the Guides’ specifications (Funk, personal communication).

## Discussion

As advocates attempt to make EBP synonymous with “value” in system-level monitoring, there is a significant need to identify and validate low-burden, high yield indicators of provider quality and effectiveness. In Washington State, we began with a systems-level perspective, working from the assumption that few if any additional resources would be available to monitor the use of EBP using external experts. As the state was already requesting that EBP be reported through session-level codes, and billing codes are already subject to potential auditing for quality, our organization focused on defining the requirements for EBP reporting and the standards for EBP documentation in routine treatment plans and progress notes. This requires minimal upfront training on how to document practice. All other reporting functions are integrated into existing, routine procedures. Integrating this reporting into routine documentation, if reporting is found to be valid, presents some significant advantages. First, EBP use is reported per session and provides a granular source of information about actual practices being used in session. Using this method, it is possible to calculate the number of sessions that used either proprietary EBPs or clinical practices within a treatment family for individual therapists, between therapists, between agencies and for the healthcare system overall. Second, the instructions for reporting EBP orients therapists to the common elements of practice and is expected to operate as a quasi-clinical guide for reducing drift away from the most active clinical elements of treatment. Third, the integration into routine auditing and reporting is expected to help move the concept of EBP away from its status as a specialty service and become an expected part of routine quality service provision. This is a systems-level shift that requires coordination at the systems level between auditing, billing, contracting and EBP liaisons. It also requires flexibility in defining and monitoring EBP that is achieved through a focus on clinical elements as the primary focus of service monitoring.

The monitoring of fidelity to EBP practice in large healthcare systems is a large challenge that is frustrating attempts to achieve a system-level shift towards routine EBP use [5, 14, 44]. The fidelity gold standard of external observation and review is widely understood to be feasible only in systems in which EBPs are viewed as specialty, “add on,” programs within the wider system of care [45, 46]. Costs and time make this approach impractical for large scale use. While lower cost alternatives are being tested in research trials through self-report tools, [33, 47, 48] and simulated sessions via behavioral rehearsal, [49] these approaches still require additional time of therapists and/or expert consultation costs and will face similar funding and implementation challenges if proposed for system-level use. The direct measurement of client outcomes, i.e., measurement-based care, is another proposed strategy for monitoring value in children’s mental health [50]. This approach offers value in directly measuring the theoretical benefit of EBP via client improvement but will also need substantive infrastructure and culture shifts to implement [14]. Another emerging approach to monitoring value that hold promise for large scale quality monitoring is automated fidelity review through natural language processing software developed to recognize behavioral codes in psychotherapy sessions. While initial results are promising for recognizing behavioral codes, the technology is still being validated, will likely require some level of final expert review (albeit more limited), and is currently only available for nonspecific therapeutic factors (e.g., empathy, listening). This may provide a promising model of automated quality review for the future as a complement or substitute for documented or externally reviewed practice.

As noted in two articles on the Distillation and Matching Model, [33, 42] the emergence of specific protocols (e.g., Coping Cat) as the optimal unit of research analysis for evidence-based mental health advancement rather than treatment families (e.g., Cognitive-Behavioral Therapy for Anxiety) is a historical artifact, resulting from the field’s interest in demonstrating the benefits of specific therapeutic approaches against the claims that “therapy” as a broad approach was ineffective [51, 52]. Subsequent studies focused on tightly controlled protocols to ensure fidelity and generate confidence in findings [53]. As these protocols began to be implemented in real world settings, the focus on fidelity to the manualized structure remained a safeguard against drift into “usual care.” However, a number of researchers have noted the problems with limiting the definition of EBP to these proprietary programs. Tested programs are often limited to a specific presenting symptom (e.g., anxiety) which means that clinicians must be trained and supervised on multiple manuals if attempting to use EBP with all clients. Further, many clients present with complex symptoms, and clinicians must be facile with knowing and using multiple EBPs in order to develop good case plans [38]. Finally, the concept of EBP itself presents an implementation barrier for some sites that could likely be otherwise engaged by component-based approaches that allow for local adaptation and ownership [54, 55]. Using the clinical treatment families and common elements framework, the Reporting Guides establishes specific criteria for eligible training, consultation, and documentation (treatment plan, progress notes) for programs that fall within these groups.

However, the approach to monitoring EBP practice through therapist attestation and billing codes presents a number of questions about whether this type of reporting guidance increases accurate reporting compared to an external certification approach. Most significantly, there is insufficient guidance from the research literature regarding the minimally necessary consultation or supervisory supports for “good enough” practice, as most research studies are focused on comparative benefit between conditions and not whether care meets an acceptable level of quality. The literature is clear that ongoing expert consultation will significantly increase the quality of care, but as healthcare systems and states determine how to allocate scarce resources, it is also critical to know how much infrastructure will be needed for achieving quality care on a large scale. As the literature evolves, states will be in a better position to determine whether training and consultation requirements should vary by the complexity of the intervention, possibly defined as interventions developed for complex or more intense treatment needs. Building the infrastructure for communicating and translating new findings from the intervention and implementation literature, illustrated here by the Reporting Guides as one example, will accelerate the translation effort from research to policy and to practice.

## Conclusions

Feasible methods for the widespread and direct monitoring of clinical care are a high priority for state and large payer systems grappling with how to measure value and support effective healthcare services. Psychotherapy services present a challenge for routine performance monitoring as the practices used in session are not easily captured in administrative data, and deferring assessments of quality to external consultants can be costly and removes the system from being able to make direct assessments of value. Our effort to develop a set of Reporting Guidelines for reporting EBP in routine clinical care attempts to solve some of the challenges related to providers’ willingness to attest to their practice. Some of the hoped for secondary benefits include building a common language about effective practice among actors in policy, research and treatment communities and opening the field for high quality training on common elements while continuing to support proprietary approaches. Current efforts are underway to systematically gather data on the feasibility and acceptability of the Reporting Guides and to plan for validation and implementation studies to understand how therapists’ and agencies’ clinical and documentation practices align with the assumptions in the guidelines. We plan to use this information to improve the Reporting Guides as well as inform the field about whether this approach can encourage the more rapid translation of science into practice and improve treatment outcomes for youth.

## Abbreviations

EBP: evidence-based practice
CFIR: Consolidated Framework for Implementation Research
CBT: cognitive behavioral therapy

## References

[1] Costello EJ, Mustillo S, Erkanli A, Keeler G, Angold A (2003). Prevalence and development of psychiatric disorders in childhood and adolescence. Arch Gen Psychiatry.

[2] AHRQ (2015). New AHRQ-funded centers to study health systems and their efforts to disseminate patient-centered outcomes research.

[3] Bruns EJ, Kerns SEU, Pullmann MD, Hensley SW, Lutterman T, Hoagwood KE (2016). Research, data, and evidence-based treatment use in state behavioral health systems, 2001–2012. Psychiatric services (Washington, DC).

[4] Hoagwood K, Essock S, Morrissey J, Libby A, Donahue S, Druss B, Finnerty M, Frisman L, Narasimhan M, Stein B (2016). Use of pooled state administrative data for mental health services research. Adm Policy Ment Health Ment Health Serv Res.

[5] Raghavan R, Bright CL, Shadoin AL (2008). Toward a policy ecology of implementation of evidence-based practices in public mental health settings. Implement Sci: IS.

[6] Askim J, Johnsen Å, Christophersen K-A (2016). Factors behind organizational learning from benchmarking: experiences from norwegian municipal benchmarking networks. Public Adm Res Theory.

[7] Kazak AE, Hoagwood K, Weisz JR, Hood K, Kratochwill TR, Vargas LA, Banez GA (2010). A meta-systems approach to evidence-based practice for children and adolescents. Am Psychol.

[8] McHugh RK, Barlow DH (2010). The dissemination and implementation of evidence-based psychological treatments: a review of current efforts. Am Psychol.

[9] Spoth R, Rohrbach LA, Greenberg M, Leaf P, Brown CH, Fagan A, Catalano RF, Pentz MA, Sloboda Z, Hawkins JD (2013). Soc prevention Res type T: addressing core challenges for the next generation of type 2 translation research and systems: the translation science to population impact (TSci impact) framework. Prev Sci.

[10] Walker SC, Bumbarger BK, Phillippi SW (2015). Achieving successful evidence-based practice implementation in juvenile justice: the importance of diagnostic and evaluative capacity. Eval Program Plann.

[11] Wandersman A, Duffy J, Flaspohler P, Noonan R, Lubell K, Stillman L, Blachman M, Dunville R, Saul J (2008). Bridging the gap between prevention research and practice: the interactive systems framework for dissemination and implementation. Am J Community Psychol..

[12] Hoagwood KE, Olin SS, Horwitz S, Cleek A, Gleacher A, Lewandowski E, Nadeem E, Acri M, Chor KHB, Kuppinger A (2014). Scaling up evidence-based practices for children and families in New York State: toward evidence-based policies on implementation for state mental health systems. J Child Clin Adolesc Psychol.

[13] Powell BJ, Beidas RS, Rubin RM, Stewart RE, Wolk CB, Matlin SL, Weaver S, Hurford MO, Evans AC, Hadley TR, Mandell DS (2016). Applying the policy ecology framework to Philadelphia’s behavioral health transformation efforts. Adm Policy Ment Health Ment Health Serv Res.

[14] Powell BJ, Beidas RS (2016). Advancing implementation research and practice in behavioral health systems. Adm Policy Ment Health Ment Health Serv Res.

[15] Rycroft-Malone J, Burton C, Bucknall T, Graham I, Hutchinson A, Stacey D (2016). Collaboration and co-production of knowledge in healthcare: opportunities and challenges. Int J Health Policy Manage..

[16] Whipp R, Pettigrew A (1992). Managing change for competitive success: bridging the strategic and the operational. Industrial and Corporate Change..

[17] Fixsen DL, Naoom SF, Blase KA, Friedman RM, Wallace F. Implementation research: a synthesis of the literature. Tamps, FL: University of South Florida, Louis de la Parte Florida Mental Health Institute, National Implementation Research Network; 2005. pp. 36–55

[18] Bogenschneider K, Corbett TJ (2011). Evidence-based policymaking: Insights from policy-minded researchers and research-minded policymakers.

[19] Prewitt K, Schwandt TA, Straf ML, National Research Council. Committee on the use of social science knowledge in public P. In: Using science as evidence in public policy. Washington, D.C.: National Academies Press; 2012.

[20] Damschroder L, Aron D, Keith R, Kirsh S, Alexander J, Lowery J (2009). Fostering implementation of health services research findings into practice: a consolidated framework for advancing implementation science. Implement Sci.

[21] Weiss CH (1977). Using social research in public policy making.

[22] Nutley SM (2007). Using evidence: how research can inform public services.

[23] Rieckmann T, Abraham A, Zwick J, Rasplica C, McCarty D (2015). A longitudinal study of state strategies and policies to accelerate evidence-based practices in the context of systems transformation. Health Serv Res.

[24] Walker SC, Lyon AR, Aos S, Trupin EW (2017). The consistencies and vagaries of the Washington State Inventory of evidence-based practice: the definition of “evidence-based” in a policy context. Adm Policy Ment Health Ment Health Serv Res..

[25] Sedlar G, Bruns EJ, Walker SC, Kerns SEU, Negrete A (2017). Developing a quality assurance system for multiple evidence based practices in a statewide service improvement initiative. Adm Policy Ment Health Ment Health Serv Res.

[26] WSIPP (2017). Updated inventory of evidence-based, research-based, and promising practices: For prevention and intervention services for children and juveniles in the child welfare, juvenile justice, and mental health systems.

[27] Beidas RS, Edmunds JM, Marcus SC, Kendall PC (2012). Training and consultation to promote implementation of an empirically supported treatment: a randomized trial. Psychiatr Serv.

[28] Giguère A, Légaré F, Grimshaw J, Turcotte S, Fiander M, Grudniewicz A, Makosso-Kallyth S, Wolf FM, Farmer AP, Gagnon MP (2012). Printed educational materials: effects on professional practice and healthcare outcomes. Cochrane Database Syst Rev..

[29] Siassakos D, Crofts J, Winter C, Weiner C, Draycott T (2009). The active components of effective training in obstetric emergencies. BJOG: An Int J Obstet Gynaecol.

[30] Coping cat (https://www.workbookpublishing.com/information.php?info_id=8). Accessed 11 Mar 2019.

[31] TF-CBT web (https://tfcbt2.musc.edu/). Accessed 11 Mar 2019

[32] Wiltsey Stirman S, Shields N, Deloriea J (2013). A randomized controlled dismantling trial of post- workshop consultation strategies to increase effectiveness and fidelity to an evidence- based psychotherapy for Posttraumatic Stress Disorder. Implement Sci: IS..

[33] Chorpita B, Daleiden E, Weisz J (2005). Identifying and selecting the common elements of evidence based interventions: a distillation and matching model. Ment Health Serv Res.

[34] England MJ, Butler AS, Gonzalez ML (2015). Psychosocial interventions for mental and substance use disorders: a framework for establishing evidence-based standards.

[35] Larsen T, Samdal O (2007). Implementing second step: balancing fidelity and program adaptation. J Educ Psychol Consult.

[36] Larsen KR, Michie S, Hekler EB, Gibson B, Spruijt-Metz D, Ahern D, Cole-Lewis H, Ellis RJB, Hesse B, Moser RP, Yi J (2017). Behavior change interventions: the potential of ontologies for advancing science and practice. J Behav Med.

[37] Rhoades BL, Bumbarger BK, Moore JE (2012). The role of a state-level prevention support system in promoting high-quality implementation and sustainability of evidence-based programs. Am J Community Psychol..

[38] Weisz JR, Chorpita BF, Palinkas LA, Schoenwald SK, Miranda J, Bearman SK, Daleiden EL, Ugueto AM, Ho A, Martin J (2012). Testing standard and modular designs for psychotherapy treating depression, anxiety, and conduct problems in youth: a randomized effectiveness trial. Arch Gen Psychiatry.

[39] Herschell AD, Kolko DJ, Baumann BL, Davis AC (2010). The role of therapist training in the implementation of psychosocial treatments: a review and critique with recommendations. Clin Psychol Rev.

[40] Jensen-Doss A, Lopez M, Hawley KM, Osterberg LD (2009). Using evidence-based treatments: the experiences of youth providers working under a mandate. Prof Psychol Res Pract.

[41] Nakamura B, Higa-McMillan C, Okamura K, Shimabukuro S (2011). Knowledge of and attitudes towards evidence-based practices in community child mental health practitioners. Adm Policy Ment Health Ment Health Serv Res.

[42] Chorpita BF, Daleiden EL (2009). Mapping evidence-based treatments for children and adolescents: application of the distillation and matching model to 615 treatments from 322 randomized trials. J Consult Clin Psychol.

[43] Dorsey S, Berliner L, Lyon AR, Pullmann MD, Murray LK (2016). A statewide common elements initiative for children’s mental health. J Behav Health Serv Res.

[44] Tabak RG, Khoong EC, Brownson RC, Chambers DA (2012). Bridging research and practice: models for dissemination and implementation research. Am J Prev Med.

[45] Beidas RS (2015). Predictors of community therapists’ use of therapy techniques in a large public mental health system. JAMA Pediatr..

[46] Kazdin AE, Weisz JR (2003). Evidence-based psychotherapies for children and adolescents.

[47] Brookman-Frazee L, Stadnick N, Roesch S, Regan J, Barnett M, Bando L, Innes-Gomberg D, Lau A (2016). Measuring sustainment of multiple practices fiscally mandated in children’s mental health services. Adm Policy Ment Health Ment Health Serv Res.

[48] Stirman SW, Gutner CA, Crits-Christoph P, Edmunds J, Evans AC, Beidas RS (2015). Relationships between clinician-level attributes and fidelity-consistent and fidelity-inconsistent modifications to an evidence-based psychotherapy. Implement Sci..

[49] Beidas RS, Edmunds J, Ditty M, Watkins J, Walsh L, Marcus S, Kendall P (2014). Are inner context factors related to implementation outcomes in cognitive-behavioral therapy for youth anxiety?. Adm Policy Ment Health Ment Health Serv Res..

[50] Lyon AR, Lewis CC, Melvin A, Boyd M, Nicodimos S, Liu FF, Jungbluth N (2016). Health information technologies-academic and commercial evaluation (HIT-ACE) methodology: description and application to clinical feedback systems. Implement Sci..

[51] Eysenck HJ (1966). The effects of psychotherapy.

[52] Smith ML, Glass GV (1977). Meta-analysis of psychotherapy outcome studies. Am Psychol.

[53] Schoenwald S, Garland A, Southam-Gerow M, Chorpita B, Chapman J (2011). Adherence measurement in treatments for disruptive behavior disorders: pursuing clear vision through varied lenses. Clin Psychol Sci Pract.

[54] Bernal G, Jimenez-Chafey MI, Domenech Rodriguez MM (2009). Cultural adaptation of treatments: a resource for considering culture in evidence-based practice. Prof: Psychol Res Pract.

[55] Lau A, Barnett M, Stadnick N, Saifan D, Regan J, Wiltsey Stirman S, Roesch S, Brookman-Frazee L (2017). Therapist report of adaptations to delivery of evidence-based practices within a system-driven reform of publicly funded children’s mental health services. J Consult Clin Psychol.

